# Dexamethasone-Loaded Nanostructured Lipid Carriers for the Treatment of Dry Eye Disease

**DOI:** 10.3390/pharmaceutics13060905

**Published:** 2021-06-18

**Authors:** Sangeeta Kumari, Madhuri Dandamudi, Sweta Rani, Elke Behaeghel, Gautam Behl, David Kent, Niall J. O’Reilly, Orla O’Donovan, Peter McLoughlin, Laurence Fitzhenry

**Affiliations:** 1 Ocular Therapeutics Research Group, Pharmaceutical and Molecular Biotechnology Research Centre, Waterford Institute of Technology, X91 K0EK Waterford, Ireland; skumari@usciences.edu (S.K.); madhuri.dandamudi@postgrad.wit.ie (M.D.); srani@wit.ie (S.R.); gbehl@wit.ie (G.B.); noreilly@wit.ie (N.J.O.); oodonovan@wit.ie (O.O.); pmcloughlin@wit.ie (P.M.); 2 Pharmaceutical Department, UC Leuven-Limburg, Campus Gasthuisberg Herestraat 49, 3000 Leuven, Belgium; ebehaeghel@novalix.com; 3 The Vision Clinic, R95 XC98 Kilkenny, Ireland; dkent@liverpool.ac.uk

**Keywords:** dry eye disease, keratoconjunctivitis sicca, dexamethasone, corticosteroid, biomarker, nanostructured lipid carrier, cell studies, translational research

## Abstract

Dry eye disease (DED) or keratoconjunctivitis sicca is a chronic multifactorial disorder of the ocular surface caused by tear film dysfunction. Symptoms include dryness, irritation, discomfort and visual disturbance, and standard treatment includes the use of lubricants and topical steroids. Secondary inflammation plays a prominent role in the development and propagation of this debilitating condition. To address this we have investigated the pilot scale development of an innovative drug delivery system using a dexamethasone-encapsulated cholesterol-Labrafac™ lipophile nanostructured lipid carrier (NLC)-based ophthalmic formulation, which could be developed as an eye drop to treat DED and any associated acute exacerbations. After rapid screening of a range of laboratory scale pre-formulations, the chosen formulation was prepared at pilot scale with a particle size of 19.51 ± 0.5 nm, an encapsulation efficiency of 99.6 ± 0.5%, a PDI of 0.08, and an extended stability of 6 months at 4 °C. This potential ophthalmic formulation was observed to have high tolerability and internalization capacity for human corneal epithelial cells, with similar behavior demonstrated on ex vivo porcine cornea studies, suggesting suitable distribution on the ocular surface. Further, ELISA was used to study the impact of the pilot scale formulation on a range of inflammatory biomarkers. The most successful dexamethasone-loaded NLC showed a 5-fold reduction of TNF-α production over dexamethasone solution alone, with comparable results for MMP-9 and IL-6. The ease of formulation, scalability, performance and biomarker assays suggest that this NLC formulation could be a viable option for the topical treatment of DED.

## 1. Introduction

Dry eye disease (DED) is one of the most prevalent eye conditions seen throughout the world. It can be broadly divided into two predominant, but often overlapping categories, of evaporative dry eye (EDE) and aqueous deficient dry eye (ADDE), with EDE being the more prevalent [1]. ADDE refers to conditions effecting lacrimal gland function while EDE is related to disorders of the lid margin such as meibomian gland disease (MGD) and ocular surface abnormalities such as mucin deficiency and contact lens-related problems.

There are multiple risk factors for DED, which as well as those already listed, include sex, race, MGD, computer use, hormone replacement therapy and medication use [1,2]. Symptoms include pain, redness, irritation, blurred vision, light sensitivity, itching and foreign body sensation and in addition to these ocular surface-related problems, there are considerable psychological effects due to interference with lifestyle and work productivity. The societal and economic burden is therefore considerable.

The lubricating system of the ocular surface consists of the moving eyelids, the meibomian glands of the lid margin, the lacrimal and accessory lacrimal glands and the tear film itself. The tear film consists of a lipid layer overlying a mucoaqueous layer. Pathologically, the defining feature of DED is tear hyperosmolarity. This in turn leads to damage and injury of the ocular surface and consequently inflammation [3]. Ultimately, this leads to goblet and epithelial cell loss and compromise of the epithelial glycocalyx and clinically this is manifest by the presence of conjunctival hyperaemia, punctate epithelial erosions, tear film instability and increased tear break up time.

Treatment of DED generally depends on the severity of symptoms but the ultimate aim is to restore homeostasis of both the ocular surface and the tear film. With longer acting strategies that include immune modulatory drugs, most sufferers can usually be managed through a combination of modification of their local environment, treatment of lid margin disease such as MGD, conservation of tears with punctum occlusion and topical treatments ranging from lubricants to short courses of topical steroid [1]. Dexamethasone (DEX) is a potent corticosteroid, marketed, for example, as a 0.1% (*w/v*) suspension as Maxidex^®^. While some of the well-known side effects of corticosteroids (cataracts, increased IOP, etc.), were outlined in a recent review by Gaballa et al., they also outlined the potential to mitigate such effects by localized, low dose administration, and highlighted the continued interest in the ophthalmic treatment potential of these drugs [4].

One of the major challenges, however, of topical ocular treatment is rapid loss of therapeutic effect due to both dilution with tears and washout through the lacrimal drainage system, hence the need for frequent administration, which in turn can paradoxically raise issues of toxicity due to systemic absorption. A safer and more promising strategy is therefore needed to mitigate these challenges and efficiently deliver the therapeutic agent to the ocular surface. An approach that has garnered significant interest is the use of nanotechnology-based ophthalmic formulations, and several nanocarriers have been developed, including nanosuspensions, liposomes, dendrimers and nanomicelles. Despite some recent additions to the market based on nanotechnology formulations [5,6], and despite extensive investigation, translation of these proof-of-concept studies to a commercial product can have many challenges, including regulatory issues, safety and in particular, scale up, as recently outlined by Gorantla et al. [7]. Nanostructured lipid carriers (NLCs), nanomaterials composed of a combination of liquid and solid lipids, are an example of a technology that lend themselves to large-scale production and the potential for extended storage stability, and so, may overcome a number of these challenges [7,8,9]. Since the seminal work on SLNs by Gasco and co-workers in the early nineties and NLC development by the team of Muller et al. almost a decade later [10,11,12], they have received considerable research attention, not least in the area of ophthalmic drug delivery [13,14]. Using many approaches to optimize the materials, from simple empirical methods to extensive statistical experimental design, researchers have demonstrated that with judicious component selection, NLCs can be optimized to have specific properties suitable for drug delivery [15,16,17]. In the current study cholesterol-Labrafac™ lipophile-based nanostructured lipid carriers (CHLF-NLCs) were prepared to encapsulate dexamethasone. Given the widespread applicability of DEX as a therapeutic, it has been investigated for use by a number of researchers for incorporation into NLCS [18,19,20]. Considering the extensive research already carried out on the optimization of both NLCs and DEX-NLCs, in the present study, the focus is instead placed on the potential translation of NLC technologies and assessment at pilot scale production. To this end, the initial step was a rapid go/no-go optimization of a bench scale formulation, followed by scale up to pilot batch scale of 1 L (i.e., 200 × 5 mL eye drop bottles), using a high pressure homogenizer [21]. Once prepared, the certified storage stability at two storage conditions and the resultant ex vivo and in vitro impact on biological tissues were investigated. These DEX-NLCs could provide therapeutic enhancement through their potentially mucoadhesive properties, their increased residence time and drug bioavailability, while reducing lacrimal drainage and systemic delivery. Since many cytokines are signaling molecules known to play an important role in the pathogenesis of DED and increased levels of cytokines such as Interleukin-1 (IL-1), IL-6, IL-8, interferon gamma (IFNγ), and Tumor necrosis factor α (TNFα) have been previously associated with ocular inflammation and severity of DED, the present study utilized ELISA to measure the DEX-NLC impact on the regulation of a panel of secreted cytokines [22,23]. Such studies, combined with the use of a rapid pre-formulation selection phase, in vitro, ex vivo and stability studies have here been used to demonstrate a potential route to bring optimal lab scale pre-formulations, through pilot scale development, to a point of readiness for commercially relevant preclinical investigation.

## 2. Materials and Methods

### 2.1. Materials

Labrafac™ lipophile WL1349 (LF) was a gift from Gattefossé (Lyon, France). Cholesterol (CH) (≥95%) and dexamethasone (DEX) (≥98%) were purchased from Carbosynth, (Compton, UK). Acetonitrile and ethanol (analytical grade), sodium hydroxide BioUltra pellets, acetone, Tween 80, dimethyl sulfoxide (DMSO), 3-(4,5-dimethylthiazol-2-yl)-2,5-diphenyltetrazolium bromide (MTT) and fluorescein isothiocyanate (FITC) were purchased from Sigma-Aldrich (Arklow, Ireland). Orthophosphoric acid was obtained from Fisher Scientific (Dublin, Ireland). Primary human corneal epithelial cells (HCECs) (ATCC^®^ PCS-700-010) were obtained from the American Tissue Culture Collection (ATCC, Manassas, VA, USA). Fetal bovine serum, phosphate buffer saline tablets, lipopolysaccharide (LPS) from *E. coli*, dimethyl sulfoxide, Maxgel^TM^ ECM mixture, human MMP-9, IL-6 and TNF-α ELISA kits were purchased from Sigma Aldrich. Dulbecco’s Modified Eagle Medium (with high glucose, GlutaMAX™ Supplement), trypsin (with EDTA (0.25%) and phenol red), Nunc™ Glass bottom dishes and T75 cell culture flasks (Nunc™ Cell Culture Treated Flasks with Filter Caps) were purchased from Fisher Scientific. CELLSTAR^®^ cell culture 6, 24 and 96 well plates with lids were obtained from Cruinn Diagnostics Ltd. (Dublin, Ireland). Human recombinant LBP protein and human CD 14 was purchased from R&D Systems (Abingdon Science Park, Abingdon, UK).

### 2.2. Methods

#### 2.2.1. Formulation of Nanostructured Lipid Carriers

The development of the lab scale formulation of NLCs used the solvent diffusion approach, adapted from a previously reported method with slight modifications [24]. In brief, formulations were prepared by mixing CH and LF (and drug or fluorescent agent where applicable) at varying ratios (Table S1) to sufficient volumes of a 50:50 acetone and ethanol mixture to dissolve all material. This solution was then heated to 60–65 °C to solubilize all components. Once achieved, organic solvent was evaporated to a volume of approximately 2 mL and the remaining solution was mixed with 10 mL of a 1% (*w/v*) solution of Tween 80 in H_2_O held at the same temperature. The resultant primary nanoemulsion was sonicated using a probe sonicator (VCX 130 PB; Sonics & Materials, Inc., Newtown, CT, USA) for 5 min at 40% amplitude, followed by cooling and stirring at RT for 1 h. After formulation, the sedimentation rates were monitored over time and lab scale formulations for analysis and long term stability were sterilized with a 0.2 µm filter before use [25].

Suitable lab scale formulations were loaded with sufficient drug to make a final DEX concentration of 0.1% (*w/v*), while fluorescent NLCs for in vitro and ex vivo studies were prepared by adding coumarin-6 at 0.1% (*w/v*) instead of DEX. Pilot scale formulation batches (PSF) of the optimized lab scale formulation (LSF4) were prepared by increasing the quantities of all materials to ensure a final volume of 1 L of 0.1% (*w/v*) DEX. The so-formed primary nanoemulsion was cycled through an Avestin C5 Emulsiflex high-pressure homogenizer (HPH) from BPS Crowthorne (Dublin, Ireland), in continuous mode at 15,000 psi for 5 min or three full cycles. After homogenization, benzalkonium chloride was added to a concentration of 0.01% (*w/v*) and final sterilization was carried out using a 0.2 µm filter [25].

#### 2.2.2. Encapsulation Efficiency and Formulation Drug Retention

In all cases DEX quantitation was carried out using high performance liquid chromatography (HPLC) on an Agilent 1200 series instrument from Agilent Technologies (Cork, Ireland). Isocratic elution with mobile phase (50:50 phosphate buffer, at pH 3, and acetonitrile) at a flow rate of 1 mL/min was performed on a sample injection volume of 20 µL. The 250 × 4.6 mm 5 µm Symmetry C_18_ column (Waters, Wexford, Ireland) was held at 20 °C and a DEX calibration curve (linear range 0.001–1 mg/mL) was obtained with an R^2^ value of 0.9998.

The encapsulation efficiency of dexamethasone entrapped in all NLCs was determined by first separating unentrapped drug from entrapped drug by centrifugation at 3000 rpm for 15 min at 15 °C. A fixed volume of NLC dispersion was then diluted with mobile phase and the entrapment efficiency was calculated by the following formula:

$$\mathrm{Encapsulation}\;\mathrm{Efficiency}\;\left(\%\right)=\frac{\left(\mathrm{Total}\;\mathrm{added}\;\mathrm{drug}-\mathrm{total}\;\mathrm{free}\;\mathrm{drug}\right)}{\mathrm{Total}\;\mathrm{added}\;\mathrm{drug}}\times 100$$

Drug retention studies were carried out on the chosen lab scale formulation and final pilot scale formulation by adding 1 mL of DEX-loaded NLC dispersion (0.1% (*w/v*) DEX) to a dialysis membrane with molecular weight cut-off of 14,000 Da (Sigma Aldrich). The sealed membranes were placed in 100 mL PBS, pH 7.4, that was placed in a water bath at a temperature of 37 °C and applying a stirring rate of 200 rpm. A 1 mL sample was withdrawn and replaced with fresh PBS every 15 min for the first 2 h, every 30 min for the next 2 h and hourly thereafter during each daily 8 h period, for a total of 7 days for LSF4 and 65 h for the PSF.

#### 2.2.3. Particle Size and Zeta Potential Measurement

Particle size and zeta potential (ZP) values were determined using dynamic light scattering (DLS) with a Microtrac, Nanotrac Wave II (Haan, Dusseldorf, Germany). Analysis was performed at 25 °C with an angle of detection 180° with the heterodyne-backscatter arrangement. An undiluted 1 mL aliquot of NLC dispersion was placed in the sample cell and the FLEX software was used to analyse electrophoretic mobility (for ZP) and particle size distribution using Brownian motion. For each NLC dispersion sampled, the mean value was recorded as an average of the average of three measurements of three independent samples.

#### 2.2.4. Cytotoxicity Study

HCECs were grown in DMEM supplemented with 10% FBS, 1% L-glutamine and 1% PenStrep at 37 °C and 5% CO_2_. The cytotoxicity of LPS (0.5–100 μg/mL) and LPS complexes consisting of LPS, CD14 and LBP at a 2:1:1 concentration ratio (e.g., 10, 5 and 5 μg/mL), was determined on HCECs. Cytotoxicity for DEX and DEX-loaded NLCs was evaluated at a formulation drug concentration of 5, 10, 20, 50 and 100 μM. Blank NLCs were prepared to the same concentration of NLC material as their drug-loaded comparators. The 96 well plates were seeded with HCECs (10,000 cells/well in DMEM medium containing 10% FBS) and cultured for 24 h. After 24 h, cells were treated with the listed test formulations and incubated for 4 h [26]. After 4 h, the wells were washed three times with PBS and 100 µL of fresh media was added to each well and further incubated for 20 h. Following incubation, 15 µL of MTT dye was added to each well and incubated for 4 h. Media in the wells was then replaced with 200 µL of DMSO to solubilise formazan crystals. Upon solubilization, the absorbance was measured at 570 nm using a microplate reader.

#### 2.2.5. Cellular Uptake Study

In vitro cellular uptake of coumarin-6 encapsulated NLCs was examined using an Olympus BX51 fluorescent microscope from Olympus Life Sciences (Waltham, MA, USA). HCECs were seeded in confocal imaging dishes (Nunc™ Glass Bottom Dishes, Fisher Scientific) at a density of 5 × 10^4^ cells per dish. The coumarin-6 encapsulated NLCs were prepared as per Section 2.2.1 and 50 µL of the fluorescently-labeled NLC solution was added to the HCECs in 2 mL of medium. After 4 h of incubation at 37 °C, the coumarin-6 loaded NLC-treated medium was removed and the cells were washed with transparent, non-phenol red containing DMEM medium. The so-treated HCECs were examined under differential interference contrast (DIC) and fluorescein isothiocyanate (FITC) mode for analysis.

#### 2.2.6. Ex Vivo Corneal Surface Distribution Study

An ex-vivo distribution study was carried out on porcine cornea to further evaluate the potential NLC behavior on the corneal surface. Porcine corneas were provided from a local abattoir within an hour of slaughter and stored in PBS, 1% (*v/v*) antibiotic solution with all studies carried out within 8 h of slaughter. The corneas were excised and placed into confocal imaging dishes containing medium, where they were then exposed to coumarin-6 encapsulated NLCs. At all stages, the excised corneas were immersed in sufficient medium to maintain a hydrated state. After 4 h of incubation at 37 °C, the medium was removed, and the cornea washed. Fluorescent microscopy under DIC and FITC modes of analysis was used to determine the corneal distribution [27].

#### 2.2.7. Cytokine Profiling Using ELISA

HCECs (5 × 10^4^ cells/well) were seeded in 24 well plates and cultured for 24 h as outlined in Section 2.2.4. After the specified time, LPS complex (Section 2.2.4) was added to each well, except for the media and cell controls, to induce inflammation. Cells were washed with media and treatments: DEX and DEX-loaded NLCs (both at a DEX concentration of 5, 10, 20, 50 and 100 μM), as well as blank NLCs prepared to the same concentration of NLC as DEX-loaded NLCs (as outlined in Section 2.2.4), were added to HCECs, after 6 h of inducing inflammation. These treatments were left for 24 h to act on inflammation-induced HCECs and the media in the wells was centrifuged and supernatants containing cytokines were stored in −80 °C until further analysis. The expression of cytokines in different conditions was analyzed using MMP-9, TNF-α and IL-6 ELISA kits, as per manufacturer protocols. Excel was used to calculate p-values generated using students t-test, where *p* < 0.05 was taken as statistically significant.

#### 2.2.8. Formulation Stability Studies

A certified stability storage facility (Q1 Scientific, Waterford, Ireland) was used to carry out stability studies on the sterilized NLC lab and pilot scale formulations containing BAK. Samples were stored at two different storage conditions: C1, 2–8 °C, and C2, 25 °C/60% relative humidity (RH) over a 24-week period. Pilot scale samples (PSF, Section 2.2.1) were tested weekly for the first 4 weeks and then monthly thereafter, while lab scale samples (LSF4, Table S1) were tested every 4 weeks. The samples were analyzed for particle size by DLS to check for changes in the size of NLCs and by HPLC to assay the drug content. Sample details and their respective storage conditions are presented in Table 1.

## 3. Results and Discussion

### 3.1. NLC Pre-Formulation Optimisation

The focus of this work was the development of a stable, potentially commercialisable nanomaterial formulation, prepared at a pilot scale, for the treatment of DED using NLCs to increase bioavailability and target the release of the active pharmaceutical [7,8]. As highlighted in the introduction, considerable work has been carried out by many researchers in the area of NLC development. Garrigue et al. recently outlined the importance of lipid-based technologies in an extensive review and NLCs are suitably well placed in terms of biocompatibility and potential drug encapsulation for such purposes [28]. However, as discussed by Narang et al. nanotechnological approaches have been extensively investigated for drug delivery purposes for almost fifty years, but with little comparative commercial success [29]. Some of the reasons for this include scale up, quality control and the navigation of regulatory concerns [14,30,31].

Whatever the ultimate approach to overcome these challenges, once the materials have been chosen, the first step in product development must begin at the laboratory or “pre-formulation” scale [7]. In the present study, the choice of lipids was based on both the solubility of the drug in the heated lipids, and the homogenous behavior of the lipids on heating, with homogenous mixtures leading to more monodisperse size ranges [32,33]. Labrafac™ (LF) was further chosen to increase bioavailability and enhance ocular penetration [34]. Tween 80 was chosen as the non-ionic surfactant due to its solubility in water and extensive use in NLC formulation and drug delivery applications [9,24,32,35]. Using the selected materials, a range of formulations, LSF1–16 (Table S1), were chosen based on the prior report by Emami et al. to empirically determine the most suitable formulations to bring forward to pilot scale [24]. Selection of the lab scale formulation to bring forward to pilot scale was carried out in a number of stages, designed to achieve the study’s objective of rapid go/no go selection. To this end, the parameters used to select material for further investigation in the first stage were a particle size below 200 nm and zeta potential between ~10–30 mV [8,9,24,36]. Formulations LSF6–10, i.e., those that contained an excess of liquid lipid above a factor of five, were immediately eliminated from selection as their particle size were all >200 nm.

The impact of the ratio of liquid to solid lipid on particle size was to be expected and has been attributed to a change in the dispersion viscosity and coalescence of the nanomaterials at high lipid concentrations [37,38]. Such an increase was in contrast to the work of Emami et al. where they observed a decrease in particle size with an increase of oleic acid with respect to their solid lipid, cholesterol, and the work of Lason et al. who saw a decrease in particle size with increased solid lipid ratios [17,24]. However, the work of Pinto et al. observed both an increase and a decrease, depending on the ratio of solid to liquid lipid and they highlight the importance of the ratio of saturated to unsaturated fatty acids [39]. Such differing results between experimental reports demonstrate the importance of careful design when specific properties are required but that varying the lipids, the ratios and the surfactant will all have an effect that can best be determined by a carefully executed DOE [32,40,41].

In the present study, the next stage of elimination was suspension stability. In this instance LSF11–16, i.e., those with an excess of the solid lipid sedimented after as little as 2 h and as such, were considered unsuitable for further experimentation (Figure S1b). As such, LSF1–5 were identified as being suitable to move forward to the next stage—DEX incorporation. Table S2 outlines the results in the five potential pre-formulation options. Of these particles, LSF5 was eliminated as the particle size was over 300 nm and LSF2 was eliminated as it was now seen to rapidly sediment (Figure S1a). Similarly to Emami et al. our drug-loaded particles were larger than those without drug, which they attributed to incorporation of the drug into the NLC matrix, stating that “the most effective factor on particle size related to the drug content” [24]. While DEX-loaded LSF4 increased in size to 251.1 nm with a PDI of 0.23, the ZP remained high at +26.7 mV and it was seen to remain stable in suspension with no sedimentation for 2 months. The encapsulation efficiency at this stage was >99% and this was the final go/no go selection criterion.

As mentioned, the key aim of this work was to rapidly select a suitable formulation based on a number of go/no go parameters and from there focus on the feasibility of scaling up and the resultant stability. To that end, of the initial pre-formulations, LSF4 was chosen as the lab scale formulation for further testing.

Adjusting the manufacturing process to include sterilization with a 0.2 µm filter reduced the particle size to 37.2 nm, though with a standard deviation of 1.46 and a reduced zeta potential to +8.4 mV, and a PDI of 0.11. Such a zeta potential could be expected for the use of a non-ionic surfactant such as Tween 80 but as stated by Kanwar et al. the NLC suspension can remain stable even under conditions of insufficient electrostatic stabilization [42]. Preliminary cytotoxicity studies showed that both blank and DEX-loaded LSF4 had a cell viability of over 80% ranging between 2–100 µg/mL of NLC, while an entrapment efficiency over 99% was obtained. Retention of the drug in PBS at pH 7.4 was over 95% for up to 7 days. Combined with the ex vivo distribution and cell internalization studies (Figures S2 and S3), which showed both cellular internalization and corneal distribution (discussed in detail for pilot scale formulation in Section 3.2.2 and Section 3.2.3), LSF4, the 1:4 CH:LF ratio formulation, was chosen for advancement to pilot scale development and further studies.

### 3.2. Pilot Scale Batch Formulation of NLCs

The next phase towards translation for a product is the formulation at pilot scale [7,30,31]. Batches at this scale are required to provide data that will effectively predict that of the production scale product, while the ability to manufacture at this scale increases the potential for commercialization and large scale manufacture [43,44]. To this end, LSF4 was scaled up to a Pilot Scale Formulation, PSF, which contained the same ratio of materials as LSF4, but prepared at a volume of 1 L. High-pressure homogenization (HPH) is a standard method for the preparation of manufacturing or pilot scale formulation batches of lipid nanoparticles [21,44,45]. Here, the pre-emulsion is prepared in the same manner as with the pre-formulation batches but once formed, is cycled through the HPH. With the settings outlined in Section 2.2.1, the CH:LF drug-loaded NLCs, PSF, were prepared at an average size of 20.64 nm. When sterilized through a 0.2 µm filter, the particle size was reduced to an average of 19.51 nm (PDI—0.08), with an encapsulation efficiency of 99.9 ± 0.5% and a zeta potential of +9.8 mV. A more controlled sample from the optimized lab scale formulation of 37.2 nm and a PDI of 0.11 to a more monodisperse sample of lower size (19.5 nm) with a minimal SD (0.51) was to be expected from HPH formulation [7]. Cunha and co-workers and Shah et al. discuss the impact of the kinetic energy of the process and how cycling through the homogenizer can promote the breakdown of the emulsion droplets to form smaller size particles in comparison to sonication [46,47]. Pilot scale development would ultimately only be necessary for the drug-loaded NLCs, as these would be the final ‘commercial product’, however for the purpose of cytotoxicity and other in vitro analyses (Section 3.2.1 and Section 3.3), a non-drug loaded NLC is required for comparison. In the case of HPH formulation, the presence of the drug had the reverse effect than that seen in the lab scale formulation. Here, the particle size was larger and particles with two populations were prepared (32.73 ± 3.72; 89.40 ± 4.24, PDI 0.14 ± 0.06 after 0.2 µm filtration). Though not the focus of this study, it is interesting to note that again, the presence of the drug appeared to be a significant factor in particle size control.

#### 3.2.1. Cytotoxicity of PSF-NLCs on HCECs

**Figure 1:**
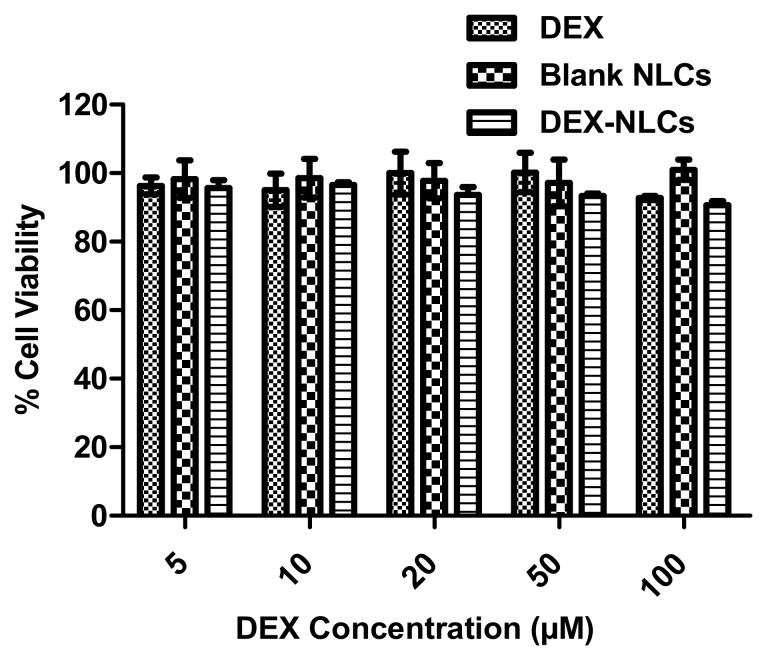
No toxicity was observed in HCECs cytotoxicity study for PSF-NLCs using DEX, blank NLCs and DEX-NLCs. Results represent *n* = 3 ± SD.

Once HPH had been proven to prepare more a controlled particle size with similar drug retention (>95% in PBS for 65 h) and appropriate encapsulation efficiency, it was necessary to confirm that that the cell tolerability had been maintained. Cytotoxicity assays were performed to assess the toxic effects of the DEX, blank and DEX-loaded PSF-NLCs on HCECs, as described in Section 2.2.4. As eye drops are quickly cleared from the ocular surface, a 4 h incubation period was considered appropriate to investigate the toxic effects of all NLCs and drug concentration. Abdelkader et al. when investigating the impact of cyclodextrin complexation on the corneal toxicity of diclofenac carried out similar studies by incubation for 4 h [26]. In the present work, the cytotoxic effect on HCECs were evaluated using a range of different concentration of DEX, blank and DEX-loaded NLCs. None of the treatments showed signs of toxicity on HCECs and demonstrated more than 90% cell viability for all treatment concentration compared to the untreated HCECs control (Figure 1) and is in agreement with previous studies testing formulations of nanoparticles [48].

#### 3.2.2. Cellular Internalization

**Figure 2:**
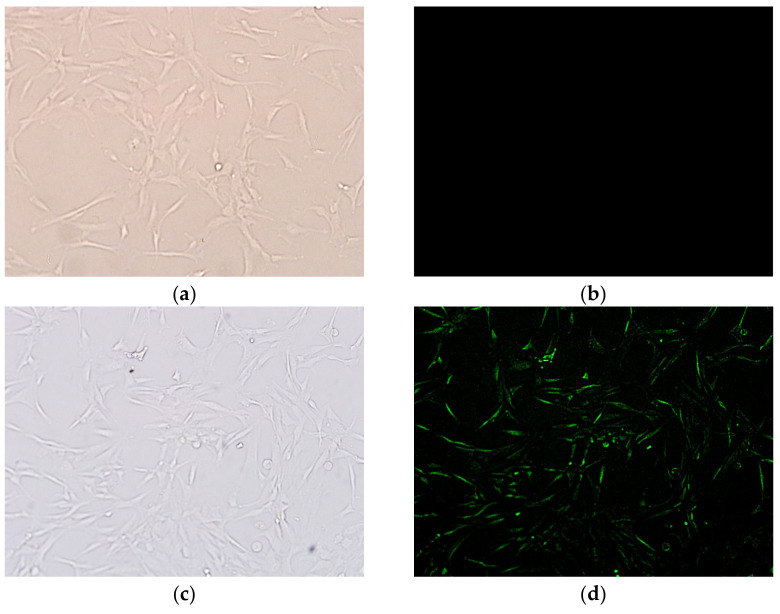
Cellular uptake analysis of coumarin-6 labelled PSF treated cells after 4 h incubation at 37 °C, 10× magnification: (**a**) Untreated control HCECs (DIC mode), (**b**) untreated control HCECs (FITC mode) showing no fluorescence, (**c**) coumarin-6 labelled PSF treated cells (DIC mode), (**d**) coumarin-6 labelled PSF treated cells (FITC mode).

It is assumed that the DEX-NLCs might be internalised by the cells via lipid membrane fusion or adhered to the HCECs monolayer [49]. As such, to ensure that cellular internalization would still take place to the required degree at this reduced particle size, a fluorescent microscope was used to characterise the uptake of fluorescent NLCs in HCECs and the preliminary internalization studies were repeated on the PSF (Figure 2). Such an experiment demonstrates the uptake of coumarin-6 loaded NLCs compared to coumarin-6 alone in solution. The fluorescence appears to be localized inside the cells (Figure 2c) suggesting that the NLCs are taken up by the HCECs, which is in contrast to Figure 2b, where no fluorescence was seen in the cells when the control coumarin-6 solution was used.

Rapalli et al. recently demonstrated intra-cellular curcumin delivery using the fluorescent properties of the curcumin loaded in their NLCs as a tag and observed fluorescent intensity peaks at both 6 and 12 h when compared to the curcumin solution controls [50]. Similarly, Yu et al. used coumarin-6 loaded NLCs in their investigation of the HCEC uptake of NLCs designed to deliver quercetin to the ocular surface, though the fluorescent material was there quantified after the cells were lysed [51]. In the present study, the focus was on a qualitative investigation of the presence of coumarin-6 loaded NLCs in HCECs when compared with untreated cells, in a method similar to that used by Nirbhavane et al. where fluorescence was used to demonstrate HCEC internalization of NLCS [27]. Once internalized within the cell, as demonstrated in Figure 2d it is hypothesized that the NLCs could release their therapeutic payload as enzyme cleavage in the endosomes, acid mediated degradation in the lysosomes, or both, would degrade the NLC components [29,52,53,54,55].

#### 3.2.3. Ex Vivo Distribution

**Figure 3:**
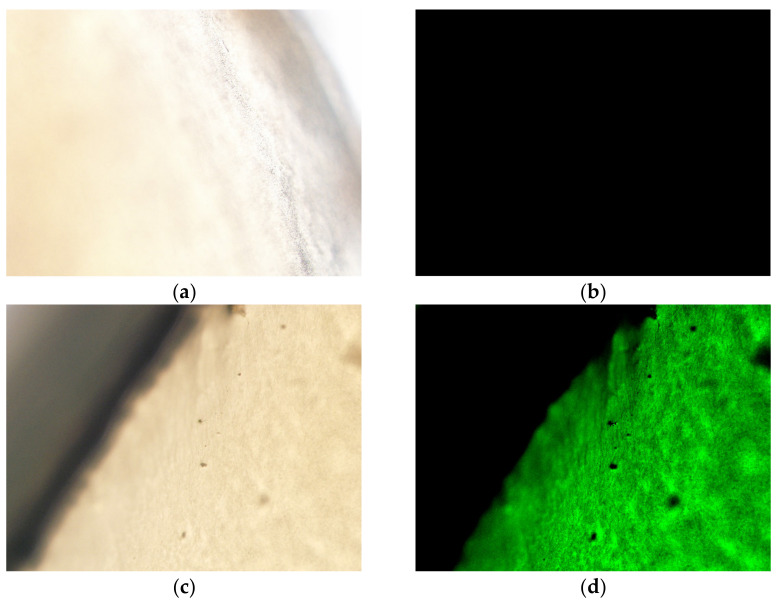
Ex vivo surface distribution analysis using porcine cornea after incubation for 4 h at 37 °C 20× magnification: All samples are planar images of the excised cornea (**a**) Untreated control cornea (DIC mode), (**b**) untreated control cornea (FITC mode), (**c**) coumarin-6 labelled PSF treated cornea (DIC mode), (**d**) coumarin-6 labelled PSF treated cornea (FITC mode). All treated corneas used 50 µL of either a 0.1% (*w/v*) coumarin-6 solution or the same volume of an NLC solution loaded with 0.1% (*w/v*) coumarin-6.

One of the primary issues with topical drops for the treatment of ocular conditions is rapid clearance of drug from the ocular surface. Mantelli and Argüeso reviewed the various functions and locations of ocular surface mucous layers, both secreted and cell-surface [56]. Cationic NLCs have the potential to take advantage of both of these layers to increase residence time on the mucosal surface and therefore extend the potential for delivery of the drug to the site of action [35]. In the present work, the corneal distribution study was carried out to demonstrate the potential bioavailability of the encapsulated drug in the ocular surface as well as the residence time of NLCs after application by proposed topical ophthalmic delivery. Figure 3 demonstrates that after an incubation time of 4 h at 37 °C, the fluorescently labelled PSF-NLCs were clearly visible on the surface of the ex vivo porcine cornea compared to the control cornea.

The mucoadhesive behavior of DEX-loaded NLCs was demonstrated for porcine cornea by Kiss et al., both with and without the use of cationic polymer coating, while Liu et al. demonstrated similar effects using coumarin-6 loaded NLCs on rabbit corneas [18,57]. In the latter study, a slight increase in retention of chitosan-coated NLCs was observed, postulated to be due to the ability of chitosan to form an interpenetration layer with the mucosal layer. In the present work, the focus is on an intermediate degree of mucoadhesion as travel through the mucous layer to the ocular epithelia may provide another route for targeted delivery [58]. Figure 3 indicates that the small particle size and increased surface area could provide sufficient bioadhesive contact with the mucosal layers and potentially provide sufficient time for the LF to further behave as a permeation enhancer and allow for internalization into the HCECs [34]. The intermediate positive charge observed for this pilot scale formulation, +9.8 mV, is postulated to be due to the presence of the Tween 80 layer. Similar effects have been observed by Hassan et al., where Tween 80 was shown to produce a “less negative” surface charge for solid lipid nanoparticles [59]. Lason et al. also highlight the transition to a positive charge when using Tween 80 alone as a surfactant for NLC formulation, recording an increase to +17.9 mV from a negative −50.1 mV when used with PlantaCare [17]. In their study on the physicochemical stimuli to modulate NLCs, Kanwar, et al. suggest that protonation of the ether linkages in Tween 80 reduced the electrical double layer of the particles and was related to increased positive values for zeta potential [42].

### 3.3. Evaluation of Cytokine Reduction in LPS-Induced Inflammation in HCECs by DEX-Loaded NLCs

Pro-inflammatory cytokines are known to promote lacrimal gland destruction (in a CD25 knockout model of Sjogren’s syndrome), goblet cell loss, keratinization of the conjunctival epithelium, as well as its apoptosis (in a dry eye mouse model), and tear film instability, all potentially aggravating DED [60,61]. Cytokines are also detected in the tear film and conjunctival epithelium obtained from DED patients, inducing inflammation of the ocular surface [60]. Matrix metalloproteinases (MMPs) can degrade the corneal extracellular matrix, which can result in epithelial cell loss and specifically, MMP-9s are produced by corneal epithelial cells in response to hyperosmolar stress, with this component having been detected in the tears of DED patients [62]. Several studies carried out in human and mice models have reported increased expression of cytokines including IL-6 and TNF-α on the ocular surface of dry eye [63,64,65] and IL-6 was found to be significantly increased (*p* < 0.01) in tears of patients suffering from Sjögren syndrome compared to non-Sjögren syndrome [66]. Further, both MMP-9 and IL-6 have been found to be significantly associated with the severity of symptoms in patients suffering with DED [66,67].

**Figure 4:**
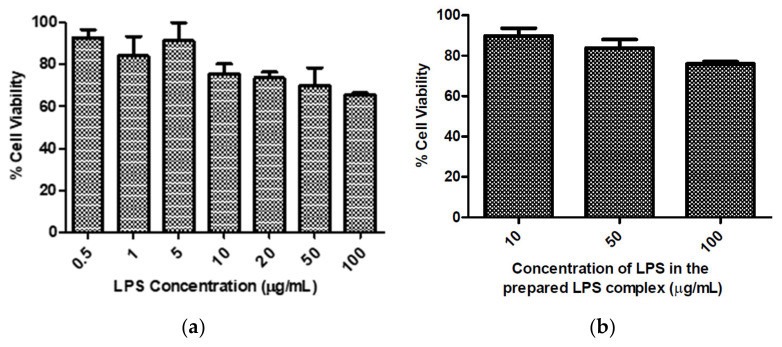
HCECs were treated with (**a**) range of different concentration of LPS and (**b**) LPS complex for 24 h, where the values on the x-axis are the concentration of LPS in the LPS complex prepared as per Section 2.2.4. Viability of untreated cells were taken as 100%. Viability of the cells decreased with increasing concentration of LPS. Results represent *n* = 3 ± SD.

LPS has been shown to induce cytokine expression and in order to evaluate the role of DEX and DEX-loaded NLCs on pro-inflammatory cytokines, it was first necessary to determine the concentration of LPS necessary to induce an inflammatory response [68]. Initially, various concentrations of LPS (0.5–100 µg/mL) were screened for cytotoxicity on HCECs. The cell viability was found to decrease with increasing LPS concentration (Figure 4a), similarly to Vantaku et al. who also reported that LPS induced cell death in HCECs in a dose-dependent manner [69].

Screening experiments with LPS alone (up to 100 µg/mL) demonstrated that this did not induce sufficient inflammation, i.e., the cytokines were not in the detectable range when using the MMP-9 ELISA kit; and as increased concentrations of LPS demonstrated increased HCEC cytotoxicity (Figure 4a), higher concentrations were not investigated further. Hydrophobic lipid A is the toxic element of LPS, which triggers the inflammation response through Toll-like receptors. Addition of CD14 and LBP catalyses the transfer of LPS to these receptors, which ultimately increases the expression of cytokines [70]. LPS in combination with CD14 and LBP (an “LPS complex”) was also used to induce an inflammatory response in HCECs by Erdinest et al., where they observed an increase in cytokine secretion compared to the use of LPS alone [65].

**Figure 5:**
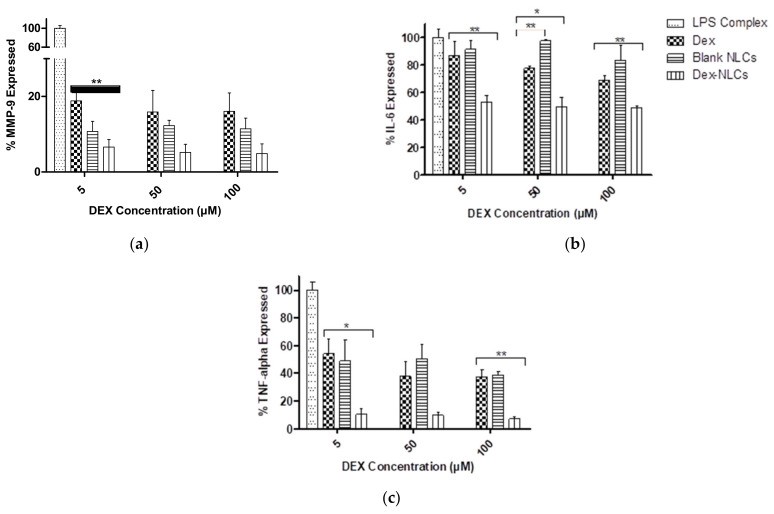
DEX, blank-NLCs and DEX-NLCs reduced inflammatory cytokines (**a**) MMP-9 (**b**) IL-6 and (**c**) TNF-α in LPS-complex induced inflammatory response compared to LPS complex (taken as 100%). * *p* ≤ 0.05, ** *p* ≤ 0.01. Note: “DEX concentration” indicates the concentration of dexamethasone in the DEX solution or DEX-loaded NLCs; blank NLC concentrations were prepared as per Section 2.2.7.

In the present work, a similar LPS complex was used to induce inflammation and cytokine secretion. LPS complex containing LPS (10 µg/mL), CD14 (5 µg/mL) and LBP (5 µg/mL) in a 2:1:1 ratio was found to be optimum for inducing inflammation in HCECs and demonstrated appropriate cytotoxicity profiles at the required concentration ranges (Figure 4b). To investigate the in-vitro anti-inflammatory activity of PSF DEX-NLCs, the expression of inflammatory cytokines (MMP-9, TNF- α and IL-6) in LPS complex-induced inflammatory response in HCECs was analysed (Figure 5).

The results (Figure 5) suggest that pure drug (DEX) and DEX-NLCs displayed an anti-inflammatory effect by lowering the expressions of cytokines. The results for DEX were expected as DEX is a very potent, FDA approved therapy for DED [67]. In all cases DEX-loaded NLCs significantly reduced the expression of all three cytokines (MMP-9, IL-6 and TNF-α) when compared to the LPS complex (*p* < 0.05). In Figure 5a, it can be seen that MMP-9 was reduced in DEX, blank-NLCs and DEX-NLCs (*p* < 0.01 in DEX-loaded NLCs compared to DEX at 5 µM concentration) compared to LPS complex treated HCECs. MMP-9 secretion was decreased as the concentration of DEX and DEX-NLCs was increased. Pflugfelder et al. also reported a significant decrease in mRNA and protein level of MMP-9 in alkali burn and desiccating stress model when treated with topical 0.1% dexamethasone four times per day [67].

When investigated in the present study, blank NLCs did not significantly regulate the expression of IL-6 compared to the LPS complex treated HCECs but IL-6 was found to be significantly reduced with increasing concentration of both DEX and DEX-NLCs (*p* < 0.05) (Figure 5b). Further, Figure 5c demonstrates that DEX and DEX-NLCs significantly reduced the TNF-α in a dose dependent manner (*p* < 0.05 and *p* < 0.01 in 5 µM and 100 µM DEX-loaded NLCs respectively compared to DEX at similar concentrations). Although blank NLCs also reduced TNF-α, this was not in a dose dependent manner compared to the LPS complex treated control. Topical DEX treatment was also found to significantly reduce both IL-6 and MMP-9 in a combined alkali ocular burn and desiccating stress murine model at both 2- and 5-days post injury [71]. DEX treatment was also seen to significantly reduce (*p* < 0.001) both IL-6 and TNF- in LPS-complex induced inflammatory response in HCECs [65]. In the present study reduced cytokine secretion (MMP-9, IL-6 and TNF-α) was observed when treated with blank NLCs compared to LPS complex treated HCECs. This could be due to presence of cholesterol in blank and DEX-loaded PSF-NLCs that makes the cell membrane rigid and decreases the permeability, thereby affecting the activation of surface receptors by LPS complex [72,73,74]. Due to blocking of the receptor by cholesterol, there might be further reduction in expression of cytokines when treated with blank and DEX-loaded PSF-NLCs. However, DEX-loaded PSF-NLCs showed further suppression of cytokine expression (MMP-9, IL-6 and TNF-α) when compared to blank NLCs due to the efficiency of DEX treatment in DEX-loaded PSF-NLCs [75].

### 3.4. Storage Stability

**Figure 6:**
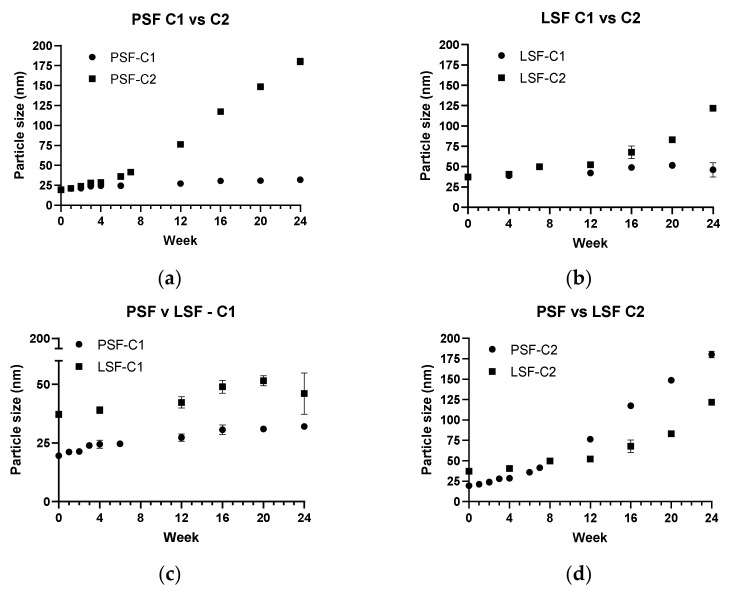
Particle size vs. number of weeks for both lab and pilot scale formulations at both storage conditions, C1 and C2; (**a**) PSF at both C1 and C2; (**b**) LSF at C1 and C2; (**c**) PSF and LSF at C1; (**d**) PSF and LSF at C2.

A further to step towards the potential commercialization of such technologies, and their ultimate use by people living with these conditions, is an investigation of the long-term storage of these materials [76]. Kanwar et al. discuss the tendency of aqueous dispersions of lipid nanomaterials to lose physical stability and as such extended periods of study should be considered [42]. Increasingly, researchers are extending this study past one or two weeks at 4 °C in a laboratory setting and as such, in the present work, two storage conditions were investigated, 2–8 °C (Condition 1, C1) and 25 °C/60% RH (Condition 2, C2) [9,37,46,50]. In order to investigate the impact of the more controlled HPH formulation, LSF4 was also stored at both temperatures. A certified storage facility was employed for this work as pharmaceutical formulations would be required to undergo such certified storage. With sampling points every week for the first four weeks and monthly thereafter, the results are presented in Figure 6.

While it is convenient to use ultrasonication at the lab scale, industrial needs would require the use of HPH for large scale manufacturing [17]. Figure 6 demonstrates that the method of nanoparticle preparation, i.e., HPH vs. sonication, at pilot vs. lab scales, respectively, had an impact on the particle size. It can be seen from Figure 6 that the degree of size change was similar for LSF and PSF at C1 and C2 but that the difference when each formulation was compared to itself was much higher for PSF. The trends observed for particle size were similar for both in that the particle size remained below 60 nm in both batches for C1 but started to increase dramatically after 3 months at C2, 25 °C/60% RH.

The largest increase in size was for PSF-C2 where the particle size increased from 21.21 ± 0.11 nm (PDI, 0.075), after one week of storage, to 180.2 ± 4.4 nm (PDI, 0.10 ± 0.006), after 6 months at this temperature. Given the increase in both size and sedimentation at this storage condition, it is likely that storage at this temperature for longer than 3 months would not be appropriate. The relative stability for up to 3 months at both temperatures was expected as Liu et al. also observed this in their NLCs at 4 °C [9]. Similar to the present work, where after 3 months the particle size was increased, Cunha et al. also observed a similar increase of 300% for their NLCs at 20 °C [46]. In all cases, the quantity of drug retained in the NLCs prepared at a pilot scale were seen to be above 99% and zeta potential remained steady (see Figure S4). Further, although not carried out in a certified stability storage facility, an 18-month study was carried out at 4 °C and demonstrated minimal size change (24.59 ± 2.66, PDI 0.55 ± 0.12), high drug content (>99%) and suitable cell tolerability (>80%).

## 4. Conclusions

Nanostructured lipid carriers and solid lipid nanoparticles have been studied for over two decades to develop novel drug delivery devices. With myriad studies demonstrating their suitability, it appears to be clear that careful design of each formulation is required. As the research community directs itself closer to translational research and the investigation of scaling up these technologies, the present study has utilised a rapid go/no go set of parameters to select an optimum pre-formulation batch to move forward to pilot scale formulation. Once appropriate drug content, cell tolerability, adhesion/internalisation properties and stability were determined, the formulation with a CH:LF ratio of 1:4 was chosen for scale up. PSF was prepared with the same ratio to 1 L of solution with 0.1% (*w/v*) dexamethasone. PSF had 99.6 ± 0.5% EE, a particle size of 19.51 ± 0.5 nm and a zeta potential of +9.8 mv, with a PDI of 0.08. At 5 °C, the particle size increased only to 32 ± 0.96 nm (PDI, 0.10) and a zeta potential of +10.4 mV, with no sedimentation and no increased cell toxicity after 6 months. PSF-NLCs demonstrated both cellular internalisation in HCECs and corneal distribution on ex vivo porcine cornea, suggesting a potentially increased bioavailability if used as a topical ophthalmic solution when compared to DEX solution alone. PSF DEX-NLCs were found to be more efficacious in reducing all investigated inflammatory cytokines (MMP-9, IL-6 and TNF-α) in an LPS complex induced inflammatory immune response compared to DEX alone, with the highest reduction seen in TNF-α where DEX-NLCs reduced cytokine expression by a factor of 5 over DEX alone. The combination of rapid pre-screening, high drug content, extended stability (up to 18 months at 4 °C) and impressive cytokine reduction suggest that such NLC formulations could be a potentially commercialisable therapeutic candidate for ocular inflammatory diseases like DED.

## Figures and Tables

**Table 1:** Storage and sample details for 6-month stability study at a certified storage facility.

NLC Batch	Storage Condition
LSF4-C1	2–8 °C
LSF4-C2	25 °C/60% RH
PSF-C1	2–8 °C
PSF-C2	25 °C/60% RH

## Data Availability

Not Applicable.

## Supplementary Materials

The following are available online at https://www.mdpi.com/article/10.3390/pharmaceutics13060905/s1, Figure S1: Lab scale formulations showing unsuitable sedimentation rates from (a) deflocculated (LSF1–3) and (b) flocculated (LSF11–16) samples; Figure S2: Cellular uptake analysis of coumarin-6 labelled LSF4 treated cells after 4 h incubation at 37 °C 20× magnification: (a) Untreated control HCEC cells (DIC mode), (b) untreated control cornea (FITC mode) showing no fluorescence observed, (c) coumarin-6 labelled LSF4 treated cells (DIC mode), (d) coumarin-6 labelled LSF4 treated cells (FITC mode); Figure S3: Ex-vivo distribution analysis using porcine cornea after incubation for 4 h at 37 °C 20× magnification (Section 2.2.6): All samples are planar images of the excised cornea (a) Untreated control cornea (DIC mode), (b) untreated control cornea (FITC mode), (c) coumarin-6 labelled LSF4 treated cornea (DIC mode), (d) coumarin-6 labelled LSF4 treated cornea (FITC mode) All treated corneas used 50 µL of either a 0.1% (*w/v*) coumarin-6 solution or the same volume of an NLC solution loaded with 0.1% (*w/v*) coumarin-6.; Figure S4. Representative images of the stability studies showing (a) zeta potential stability study for PSF at C1 and (b) encapsulation efficiency for PSF at C2; Table S1: Formulation components for preliminary lab scale optimization; Table S2: Initial lab scale optimization for CHLF blank and DEX-loaded NLCs (*n* = 3).
