# Supplementary material for: Dexamethasone-Loaded Nanostructured Lipid Carriers for the Treatment of Dry Eye Disease
Source: Pharmaceutics. 2021 Jun 18;13(6):905. doi: 10.3390/pharmaceutics13060905 (PMC8234689; doi:10.3390/pharmaceutics13060905)
Supplement: Supplementary file 1 [file pharmaceutics-13-00905-s001.zip › pharmaceutics-1081004-supplementary.pdf]

pharmaceutics

Supplementary Materials: Dexamethasone-Loaded Nanostructured Lipid Carriers for the Treatment of Dry Eye Disease

Sangeeta Kumari, Madhuri Dandamudi, Sweta Rani, Elke Behaeghel, Gautam Behl, David Kent, Niall J. O'Reilly, Orla O'Donovan, Peter McLoughlin and Laurence Fitzhenry

Table S1. Formulation components for preliminary lab scale optimization.

Formulation	Ratio CH:LF	CH (mg)	LF (µL)
LSF1	1:1	10	10
LSF2	1:2	10	20
LSF3	1:3	10	30
LSF4	1:4	10	40
LSF5	1:5	10	50
LSF6	1:10	10	100
LSF7	1:20	10	200
LSF8	1:30	10	300
LSF9	1:35	10	350
LSF10	1:40	10	400
LSF11	2:1	20	10
LSF12	4:1	40	10
LSF13	5:1	50	10
LSF14	10:1	100	10
LSF15	20:1	200	10
LSF16	30:1	300	10

Table S2. Initial lab scale optimization for CHLF blank and DEX-loaded NLCs (n = 3).

Formulation	Blank			DEX (1 mg/mL)		
	Size (nm)	PDI	ZP (mV)	Size (nm)	PDI	ZP (mV)
LSF1	75.60	0.02	+17.7	2732	0.68	+17.6
LSF2	75.30	0.03	+18.7	81.40	0.02	+29,2
LSF3	94.30	0.12	+20.0	161.7	0.05	+25.4
LSF4	136.0	0.09	+24.0	251.1	0.23	+26.7
LSF5	175.9	0.26	+23.5	306.0	0.33	+25.4

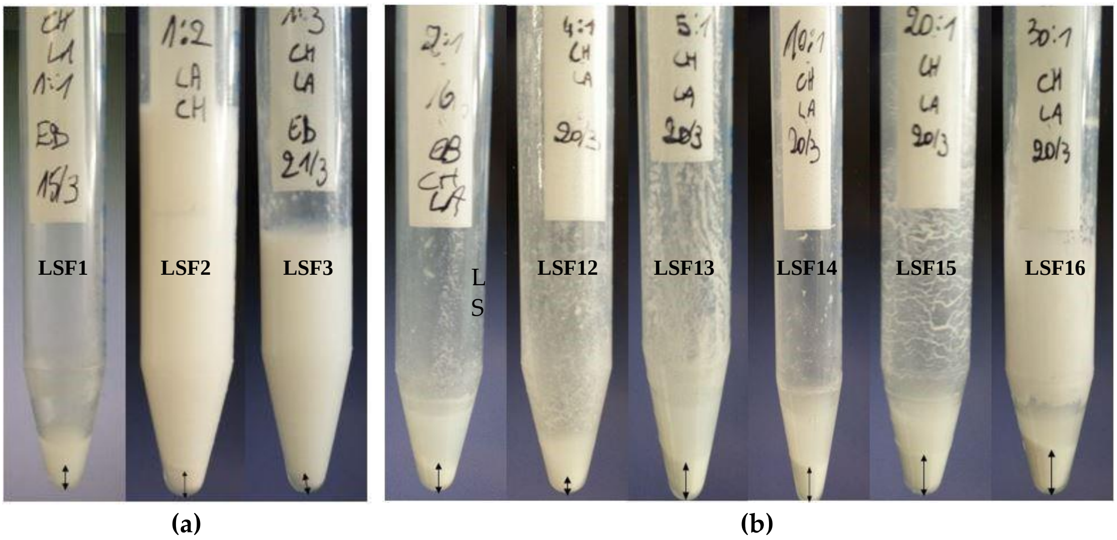

Figure S1. Lab scale formulations showing unsuitable sedimentation rates from (**a**) deflocculated (LSF1-3) and (**b**) flocculated (LSF11-16) samples.

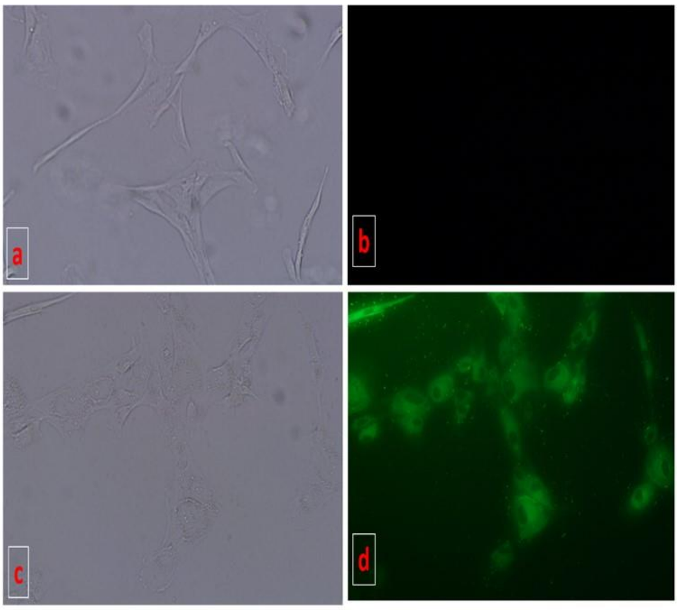

Figure S2. Cellular uptake analysis of coumarin-6 labelled LSF4 treated cells after 4 h incubation at 37 °C 20× magnification: (**a**) Untreated control HCEC cells (DIC mode), (**b**) untreated control cornea (FITC mode) showing no fluorescence observed, (**c**) coumarin-6 labelled LSF4 treated cells (DIC mode), (**d**) coumarin-6 labelled LSF4 treated cells (FITC mode).

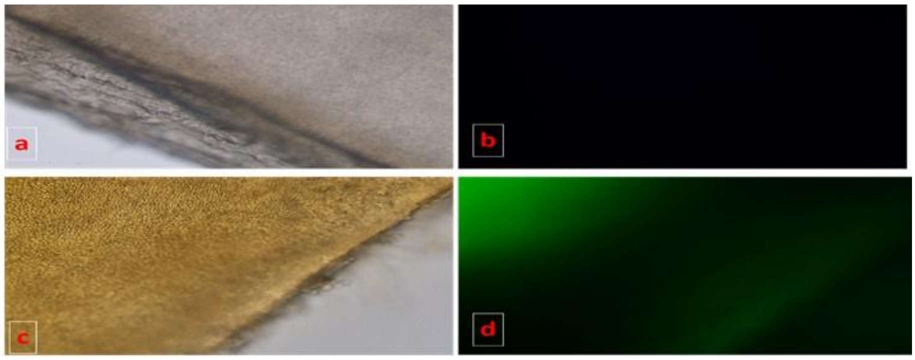

Figure S3. *Ex-vivo* surface distribution analysis using porcine cornea after incubation for 4 h at 37 °C 20× magnification (Section 2.2.6): All samples are planar images of the excised cornea (**a**) Untreated control cornea (DIC mode), (**b**) untreated control cornea (FITC mode), (**c**) coumarin-6 labelled LSF4 treated cornea (DIC mode), (**d**) coumarin-6 labelled LSF4 treated cornea (FITC mode) All treated corneas used 50 μL of either a 0.1% (*w*/*v*) coumarin-6 solution or the same volume of an NLC solution loaded with 0.1% (*w*/*v*) coumarin-6.

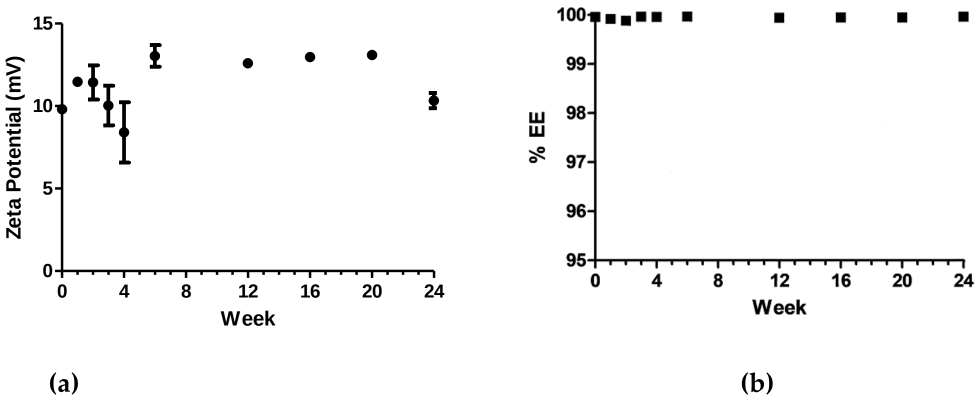

Figure S4. Representative images of the stability studies showing (**a**) zeta potential stability study for PSF at C1 and (**b**) encapsulation efficiency for PSF at C2.
